# Clinical Value of Core Needle Biopsy as a Second-Line Approach After Non-Conclusive Fine-Needle Aspiration in Thyroid Nodules: A Paired Analysis

**DOI:** 10.3390/diagnostics16071104

**Published:** 2026-04-07

**Authors:** Vladan Markovic, Slobodanka Mitrovic, Tijana Maksic, Irfan Corovic, Marija Sekulic, Mladen Maksic, Vesna Grbovic

**Affiliations:** 1 Department of Radiology, Faculty of Medical Sciences, University of Kragujevac, Svetozara Markovica 69, 34000 Kragujevac, Serbia; drjack.vm@gmail.com; 2 Department of Pathology, Faculty of Medical Sciences, University of Kragujevac, Svetozara Markovica 69, 34000 Kragujevac, Serbia; smitrovic@fmn.kg.ac.rs; 3 Department of Pediatrics, Faculty of Medical Sciences, University of Kragujevac, Svetozara Markovica 69, 34000 Kragujevac, Serbia; tijanaveljkovic96@gmail.com; 4 Center for Molecular Medicine and Stem Cell Research, Faculty of Medical Sciences, University of Kragujevac, Svetozara Markovica 69, 34000 Kragujevac, Serbia; ira.corovic@gmail.com; 5 Department of Hygiene and Ecology, Faculty of Medical Sciences, University of Kragujevac, Svetozara Markovica 69, 34000 Kragujevac, Serbia; msekulic82@gmail.com; 6 Department of Internal Medicine, Faculty of Medical Sciences, University of Kragujevac, Svetozara Markovica 69, 34000 Kragujevac, Serbia; 7 Department of Physical Medicine and Rehabilitation, Faculty of Medical Sciences, University of Kragujevac, Svetozara Markovica 69, 34000 Kragujevac, Serbia; grbovicvesna72@gmail.com

**Keywords:** thyroid nodules, fine-needle aspiration biopsy, core needle biopsy, Bethesda system, ACR TI-RADS, diagnostic accuracy

## Abstract

**Background:** Fine-needle aspiration biopsy (FNAB) is the standard initial diagnostic procedure for thyroid nodules; however, a considerable proportion of results are non-diagnostic or indeterminate, often requiring repeat procedures and delaying management. Core needle biopsy (CNB) has been proposed as a second-line option. This study evaluated the frequency of non-conclusive FNAB and CNB results and assessed the diagnostic contribution of CNB in nodules with initially non-conclusive FNAB findings. **Methods:** A retrospective–prospective study was conducted between 2019 and 2025 at a tertiary referral center, including 434 thyroid nodules. Ultrasound risk stratification followed ACR TI-RADS criteria. FNAB was performed in 430 nodules, and CNB in 85 nodules, including 82 evaluated by both methods. Biopsy results were classified according to the Bethesda system as conclusive or non-conclusive. Paired comparisons were analyzed using the McNemar test, and associations with ultrasound risk were assessed. **Results:** FNAB produced non-conclusive results in 56.5% of cases, compared with 23.5% for CNB. In paired analysis, 53.7% of nodules with non-conclusive FNAB were reclassified as conclusive after CNB (*p* < 0.001). CNB significantly distinguished benign from malignant lesions, unlike FNAB. Hypoechogenicity, irregular margins, and punctate echogenic foci were independent predictors of malignancy. Minor complications were more frequent after CNB, while major complications were rare in both groups. **Conclusions:** CNB improves diagnostic yield when used as a second-line procedure in nodules with non-conclusive FNAB findings. Selective use in higher-risk nodules may reduce repeat procedures and facilitate more structured clinical management.

## 1. Introduction

Thyroid nodules (TNs) represent a common clinical finding, with prevalence varying considerably depending on the diagnostic modality applied [1,2]. Although most nodules are benign, a smaller proportion may represent malignancy, and several risk factors—including hormonal influences and patient-related characteristics—have been associated with the development of differentiated thyroid carcinoma. Accurate differentiation between benign and malignant lesions is therefore essential for appropriate clinical management and therapeutic planning [3,4].

Ultrasound (US) is the primary diagnostic tool for the evaluation and follow-up of thyroid nodules and plays a central role in malignancy risk assessment [5,6]. The American College of Radiology Thyroid Imaging Reporting and Data System (ACR TI-RADS) provides standardized risk stratification based on specific sonographic features and assists in identifying nodules that require biopsy. However, imaging findings alone are insufficient to establish a definitive diagnosis, and tissue sampling remains necessary in nodules meeting established criteria [7,8].

Fine-needle aspiration biopsy (FNAB) is the standard first-line method for cytological evaluation of thyroid nodules. Despite its widespread use and high diagnostic value, a substantial proportion of FNAB results fall into non-diagnostic or indeterminate Bethesda categories (I, III, and IV), which may complicate clinical decision-making [9,10]. Such findings often lead to repeated biopsies, prolonged diagnostic pathways, and, in certain cases, surgical intervention without definitive preoperative confirmation of malignancy. Core needle biopsy (CNB) has been introduced as an alternative or complementary diagnostic method, particularly in nodules with previously non-conclusive FNAB findings. By providing a larger tissue sample with preserved architecture, CNB may enable more reliable histological assessment and potentially reduce the frequency of indeterminate results [11,12]. According to current guidelines, core needle biopsy is not considered a routine diagnostic procedure for thyroid nodules and should be reserved for experienced centers with appropriate expertise and immediate availability of treatment in case of complications [13,14].

The aim of this study was to assess the frequency of non-conclusive findings obtained by FNAB and CNB and to evaluate the diagnostic contribution of CNB in thyroid nodules with initially non-conclusive cytology, including paired analysis of nodules examined by both methods.

## 2. Material and Methods

### 2.1. Study Design

This study was designed as a single-center observational study with combined retrospective and prospective data collection. It was conducted at a tertiary referral institution (University Clinical Center Kragujevac) between January 2019 and September 2025. The study protocol was approved by the institutional Ethics Committee, and all participants provided written informed consent.

### 2.2. Study Population

A total of 434 adult patients of both genders (≥18 years) with thyroid nodules referred for biopsy were included in the study. Patients were evaluated and treated at the University Clinical Center Kragujevac during the study period. Clinical and imaging data were retrieved from institutional electronic medical record systems (RIS and Heliant). Pediatric patients are referred to specialized tertiary centers, and therefore CNB is not routinely performed in this population at our institution.

### 2.3. Inclusion and Exclusion Criteria

Inclusion: patients ≥ 18 years with nodules meeting the ACR TI-RADS criteria for biopsy or with a high risk of malignancy. Exclusion: patients < 18 years, pregnant women without urgent biopsy indication, febrile conditions, uncontrolled coagulation disorders, skin infections at the puncture site, or inability to cooperate.

### 2.4. Ultrasound Assessment

Ultrasound examinations were performed using high-resolution Samsung V8 devices (Samsung, Belgrade, Serbia) with linear probes (5–12 MHz). Ultrasound evaluation of thyroid nodules was performed using the ACR TI-RADS. Each nodule was assessed according to standardized criteria (composition: cystic, spongiform, mixed, or solid; echogenicity: anechoic, iso-/hyperechoic, hypoechoic, or markedly hypoechoic; shape: wider-than-tall or taller-than-wide; margins: smooth, ill defined, lobulated/irregular, or with extrathyroidal extension; echogenic foci: none or large comet-tail artifacts, macrocalcifications, peripheral/rim calcifications, or punctate echogenic foci). A specific number of points was assigned to each feature, and the total score was calculated to classify the nodule into the appropriate TI-RADS category (TR1–TR5). Based on the total score, nodules were categorized as TR1 (benign), TR2 (not suspicious), TR3 (mildly suspicious), TR4 (moderately suspicious), and TR5 (highly suspicious) [15]. The indication for biopsy was determined in accordance with ACR TI-RADS recommendations, taking into account both nodule size (greatest diameter) and the corresponding risk category. ACR TI-RADS evaluation was performed by an experienced radiologist, and each nodule was assigned the category corresponding to its ultrasound characteristics. Biopsies were performed on nodules with higher ACR TI-RADS scores or suspicious characteristics. Nodule size was measured in three planes, and orientation was determined in the transverse plane.

### 2.5. Biopsy Procedure

FNAB was performed as the initial biopsy procedure in all nodules, except in cases with suspicion of undifferentiated thyroid carcinoma, medullary thyroid carcinoma, lymphoma, or metastatic disease, in which CORE biopsy was performed as the first-line procedure. CORE biopsy was also performed following a non-diagnostic FNAB result. FNAB was carried out using 27gauge needles, with three to five passes per nodule, with or without aspiration. CORE biopsy was performed using an automated 18-gauge biopsy device, with one to two passes per nodule, preceded by a small skin incision. All procedures were conducted under local anesthesia and ultrasound guidance in sterile conditions. Manual compression was applied after the biopsy (5 min for FNAB and 15 min for CORE), followed by a control ultrasound examination within 12–24 h.

Core needle biopsy (CNB) was primarily performed as a second-line diagnostic procedure following non-diagnostic or indeterminate FNAB results. In a limited number of cases (*n* = 10), CNB was used as a first-line method. Among these, three nodules were evaluated exclusively with CNB, while in seven cases both FNAB and CNB were performed during the same session.

First-line CNB was reserved for nodules with strong clinical suspicion of specific malignancies, including medullary thyroid carcinoma, anaplastic carcinoma, lymphoma, or metastatic disease. The decision was based on ultrasound characteristics, clinical presentation, and patient medical history.

The interval between FNAB and subsequent biopsy (including CNB) was individualized based on clinical and ultrasound risk assessment. In nodules with low suspicion of malignancy, repeat biopsy was generally performed after approximately three months, whereas in high-risk nodules it was performed within a shorter interval, typically within one month, in accordance with previously published recommendations [16,17,18].

The decision to perform CNB was primarily made by the interventional radiologist based on ultrasound findings, clinical context, and prior FNAB results, with consultation with an endocrine surgeon in selected cases.

The performance of CNB depended on nodule size, location, and technical limitations of the biopsy system. In our cohort, the smallest nodule subjected to CNB measured approximately 13 mm in the largest diameter.

CNB was not performed based on a single ultrasound feature (such as nodule orientation), but rather on an integrated assessment of ultrasound characteristics, clinical context, and prior FNAB findings.

All biopsy procedures were performed by an interventional radiologist with more than 15 years of experience in ultrasound-guided percutaneous biopsies. Cytological and histopathological analyses were performed by an experienced pathologist specialized in thyroid pathology.

Biopsy was not performed routinely in nodules suspected to represent colloid goiter, but only in cases with suspicious ultrasound features meeting established biopsy criteria.

### 2.6. Cytological and Histopathological Analysis

FNAB samples were prepared using standard H&E staining and fixed in 10% formalin, while CORE samples were processed in paraffin blocks, sectioned at 3–4 μm, and stained with H&E. Cytological and histopathological evaluations of thyroid nodules were performed using fine-needle aspiration biopsy (FNAB) and core needle biopsy (CNB), respectively. All samples were analyzed by an experienced pathologist blinded to the ultrasound findings. Cytological findings were classified according to the updated 2023 Bethesda System for Reporting Thyroid Cytopathology, which includes six diagnostic categories: (I) non-diagnostic, (II) benign, (III) atypia of undetermined significance (AUS), (IV) follicular neoplasm (FN), (V) suspicious for malignancy, and (VI) malignant. Cytological findings obtained prior to 2023 were retrospectively aligned with the updated classification [16]. Each nodule was assigned the highest Bethesda category observed in its sample, allowing for standardized risk stratification and direct comparison between FNAB, CNB, and definitive cytological and histopathological outcomes.

For the purpose of analysis, Bethesda categories I, III, and IV were classified as non-conclusive, as they are associated with diagnostic uncertainty and often require additional diagnostic procedures, including repeat biopsy, molecular testing, or diagnostic surgery. The term “non-conclusive” was used to describe findings that do not allow definitive classification as benign or malignant and require further diagnostic evaluation, including Bethesda category I. In contrast, categories II, V, and VI were considered conclusive, as they generally provide sufficient information to guide clinical management. This classification is in accordance with the clinical applicability of the Bethesda system and has been supported by previous studies. This classification reflects clinical practice, where Bethesda categories III and IV are considered indeterminate and typically require additional diagnostic evaluation. Histopathological evaluation was performed in accordance with the 2022 WHO Classification of Tumours of Endocrine and Neuroendocrine Organs (5th edition) [17].

### 2.7. Measured Variables

Independent: FNAB and CORE biopsy; ACR TIRADS score.

- Dependent: number of non-diagnostic results, number of repeated biopsies, and Bethesda category.
- Confounding: sex, age, and Hashimoto thyroiditis.

### 2.8. Study Sample Size

Sample size was calculated using Student’s *t*-test for two independent samples (G*Power3), with an effect size of 0.5, α = 0.05, and power of 0.8. Based on these assumptions, the required number of participants per group was 64, allowing the detection of statistically significant differences.

### 2.9. Statistical Analysis

**Figure 1:**
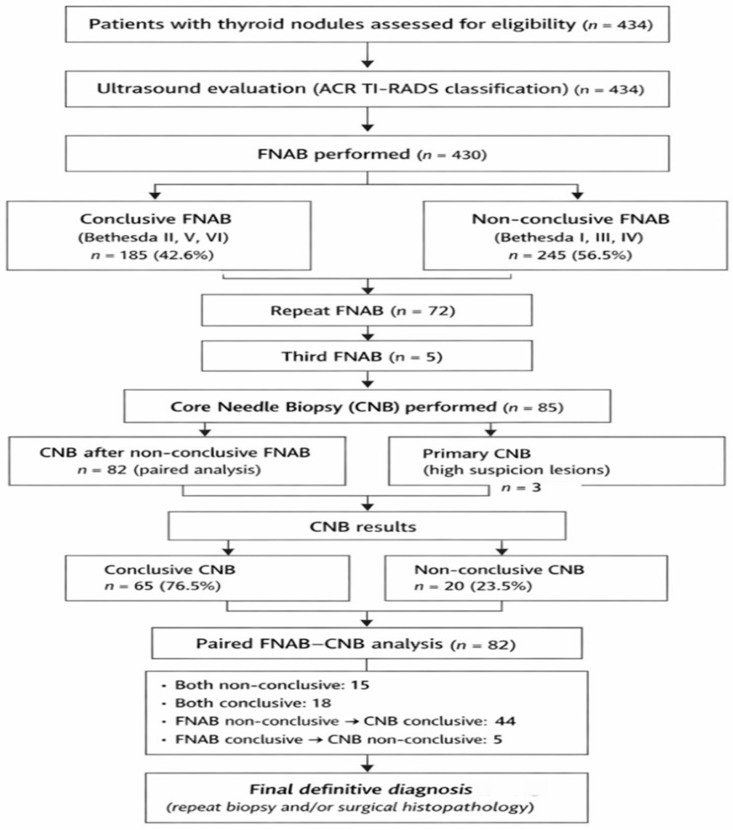
Study flowchart showing patient inclusion, diagnostic workflow, distribution of conclusive and non-conclusive biopsy results, and paired FNAB–CNB comparison.

All statistical analyses were performed using IBM SPSS Statistics software (version 21.0; IBM Corp., Armonk, NY, USA). Categorical variables are presented as counts and percentages. Between-group comparisons were performed using the χ^2^ test or Fisher’s exact test, as appropriate. The association between ordinal variables was assessed using Spearman’s rank correlation coefficient (ρ). Agreement between FNAB and CNB findings obtained from the same nodule was evaluated using the McNemar test. To identify independent predictors of malignancy, logistic regression analysis was performed. Univariate logistic regression was initially conducted for each potential predictor, and variables with *p* ≥ 0.10 were excluded. A multivariable logistic regression model was subsequently constructed using stepwise variable selection. Results are reported as odds ratios (OR) with 95% confidence intervals. Diagnostic performance was assessed by calculating sensitivity, specificity, positive predictive value, and negative predictive value using standard definitions. A two-tailed *p*-value < 0.05 was considered statistically significant. The overall patient selection process, biopsy sequence, and paired FNAB–CNB analysis are summarized in Figure 1.

## 3. Results

### 3.1. Patient and Nodule Characteristics

A total of 434 nodules were included in this study, of which 98 (22.6%) were observed in male patients and 336 (77.4%) in female patients. The mean age of all patients was 55.6 ± 14.3 years, with the youngest patient being 18 and the oldest 84 years old. The distribution of the 434 thyroid nodules revealed a skewed pattern, with a higher number of smaller nodules and a gradual decrease in frequency among larger nodules. The mean nodule size was 25.45 ± 14.16 mm, while the median was 23.00 mm. Nodule sizes ranged from 5 to 155 mm. The majority of nodules were located in the left thyroid lobe (222; 51.2%), there were fewer in the right lobe (200; 46.1%), and the least frequent location was the isthmus (12; 2.8%). Most patients had multiple thyroid nodules, observed in 286 cases (65.9%), while a solitary nodule was found in 148 cases (34.1%).

### 3.2. Ultrasound Predictors of Malignancy

The majority of nodules (352; 81.1%) were of solid structure, while spongiform nodules were the least common, observed in only 3 cases (0.7%). Regarding echogenicity, hypoechoic nodules predominated, present in 171 cases (39.5%), whereas anechoic nodules were the least frequent, observed in 9 cases (2.1%). In terms of margins, well-defined and smooth margins were seen in 351 nodules (81.3%), while poorly defined margins were the least common, found in 3 nodules (0.7%). Most nodules (297; 68.4%) were horizontally oriented (“wider than tall”), while vertical orientation (“taller than wide”) was observed in 135 nodules (31.1%). Concerning the presence of echogenic foci, 223 nodules (51.4%) showed no echogenic structures, while peripheral calcifications were the rarest, seen in 5 nodules (1.2%) (Table 1).

Based on nodule characteristics, a multivariate binary logistic regression analysis was performed to identify independent predictors of malignancy in patients with thyroid nodules. Only variables that showed a statistical significance of *p* < 0.1 in univariate analysis were included in the model. The multivariate analysis included echogenicity, margin characteristics, presence of echogenic foci and nodule size. The resulting model was statistically significant (χ^2^ = 36.755; df = 7; *p* < 0.001) and demonstrated good fit according to the Hosmer–Lemeshow test (χ^2^ = 9.791; *p* = 0.280). The Cox & Snell R^2^ and Nagelkerke R^2^ values were 0.528 and 0.708, respectively, indicating that the model explains approximately 71% of the variability in the dependent variable. In the final model, the independent statistically significant predictors were hypoechogenicity/marked hypoechogenicity of the nodule, irregular margins, and the presence of punctiform echogenic foci. Hypoechoic or markedly hypoechoic nodules showed a 22-fold higher likelihood of malignancy compared to hyperechoic and isoechoic nodules (OR = 22.321; 95% CI = 1.304–382.101; *p* < 0.05), while irregular or lobulated margins, or extrathyroidal extension, increased the risk of malignancy approximately 14-fold (OR = 14.606; 95% CI = 1.009–211.422; *p* < 0.05).

**Figure 2:**
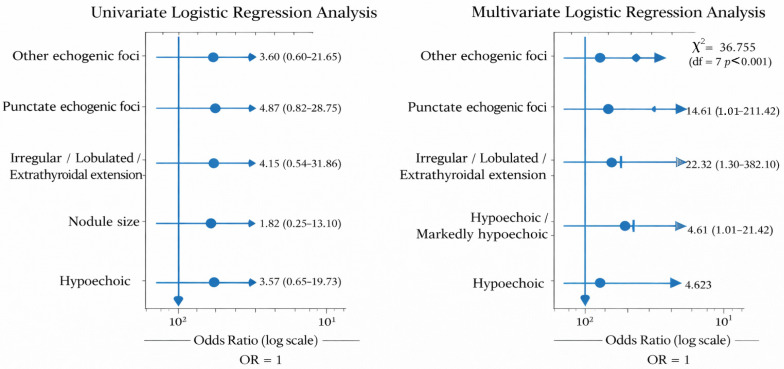
Univariate and multivariate logistic regression analyses of ultrasound predictors of thyroid malignancy.

The presence of punctiform echogenic foci increased the likelihood of a malignant finding more than 18-fold (OR = 18.562; 95% CI = 1.722–200.033; *p* = 0.016), as shown in Figure 2 and Table 2.

**Figure 3:**
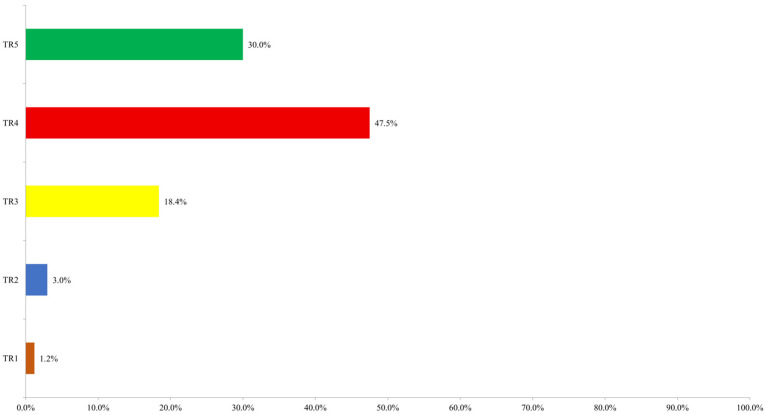
Distribution of nodules according to ultrasound ACR TI-RADS score.

According to the ultrasound ACR TI-RADS assessment, the majority of nodules (206; 47.5%) were classified as TR4, followed by TR5 with 130 nodules (30.0%) and TR3 with 80 nodules (18.4%). Fewer nodules were categorized as TR2 (13; 3.0%) and TR1 (5; 1.2%). The distribution of nodules according to ultrasound ACR TI-RADS category is shown in Figure 3.

### 3.3. FNAB Results

**Figure 4:**
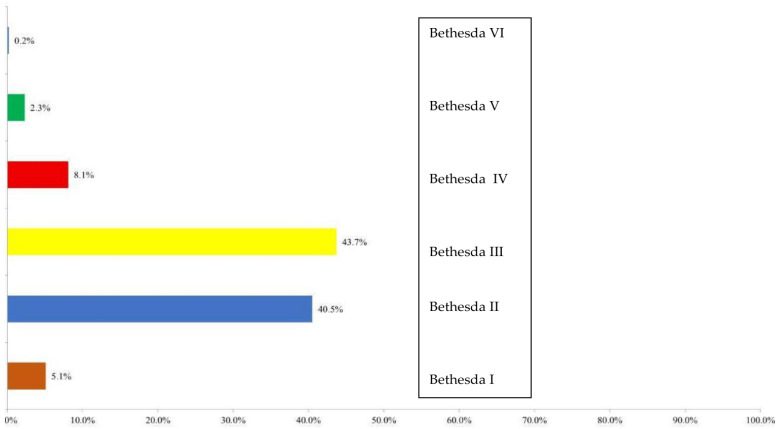
Distribution of FNAB findings according to the Bethesda system.

After the characterization of the nodules by ultrasound and classification into an ACR TIRADS category, FNA was performed. Cytological analysis of thyroid nodules performed by fine-needle aspiration biopsy (FNAB) included a total of 430 nodules. The majority of findings were classified as category III (188; 43.7%), followed by category II (174; 40.5%). Category IV was observed in 35 nodules (8.1%), category I in 22 nodules (5.1%), category V in 10 nodules (2.3%), and category VI in 1 nodule (0.2%). The distribution of FNAB findings according to the Bethesda system is shown in Figure 4.

All cytological findings were further grouped into two categories: conclusive and non-conclusive. Non-conclusive findings included Bethesda categories I, III, and IV, while conclusive findings included categories II, V, and VI. There were 245 non-conclusive findings (56.5%) and 185 conclusive findings (42.6%).

### 3.4. Correlation Between FNAB and ACR TI-RADS

**Figure 5:**
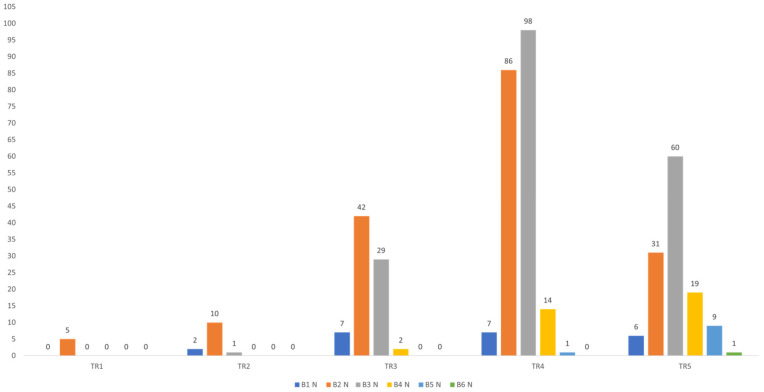
Distribution of FNAB findings according to ACR TI-RADS score.

A statistically significant association was observed between the distribution of FNAB findings and ACR TIRADS categories (Fisher’s exact test; *p* < 0.001). In Bethesda categories I and III, the most common ACR TIRADS score was TR4, with 86 findings (49.4%) in the Bethesda II category and 98 findings (52.1%) in the Bethesda III category classified as TR4. In the higher Bethesda categories IV, V, and VI, TR5 findings predominated. Specifically, in the Bethesda IV category, 19 findings (54.3%) were TR5; in Bethesda V, 9 findings (90.0%); and in Bethesda VI, the single finding (100.0%) was TR5. A statistically significant positive correlation was found between ACR TI-RADS and FNAB results (ρ = 0.315; *p* < 0.001), indicating that higher ACR TI-RADS categories are associated with higher FNAB scores, as shown in Figure 5.

### 3.5. Repeat FNAB Outcomes

Repeat FNAB analysis was performed on 72 nodules. Among these, 36 nodules (50.0%) were classified as category III, 32 nodules (44.4%) as category II, 2 nodules (2.8%) as category IV, and categories I and V were each observed in 1 nodule (1.4%). A third FNAB analysis was performed on 5 nodules, with 3 nodules (60.0%) classified as category II and 2 nodules (40.0%) as category III.

### 3.6. CNB Results

**Figure 6:**
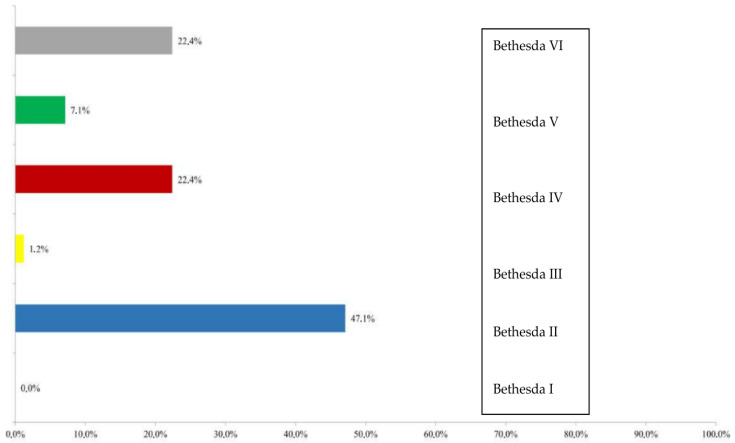
Distribution of CNB findings according to the Bethesda system.

Histopathological assessment of thyroid nodules using core needle biopsy (CNB) was performed on a total of 85 nodules. The majority of findings were classified as category II in 40 nodules (47.1%), followed by category IV in 19 nodules (22.4%) and category VI in 19 nodules (22.4%). Category V was observed in 6 nodules (7.1%) and category III in 1 nodule (1.2%). The distribution of CNB findings according to categories is shown in Figure 6.

All CNB findings were further grouped into two categories: conclusive and non-conclusive. There were 20 non-conclusive findings (23.5%) and 65 conclusive findings (76.5%).

**Figure 7:**
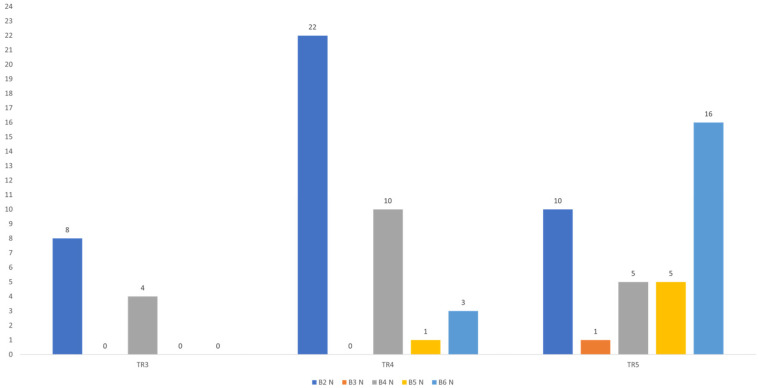
Distribution of CNB findings according to ACR TI-RADS score.

A statistically significant association was observed between the distribution of CNB findings and ACR TI-RADS categories (Fisher’s exact test; *p* < 0.001) (Table 3). In CNB categories II and IV, the most common ACR TI-RADS score was TR4, with 22 findings (55.0%) in the II category and 10 findings (52.6%) in the IV category classified as TR4. Conversely, in the higher CNB categories V and VI, the majority of samples were classified as TR5, with 5 findings (83.3%) in Bethesda categories V and 16 findings (84.2%) in VI assigned to this ACR TI-RADS score. In the Bethesda III category, only a single finding was recorded, classified as TR5. A statistically significant positive correlation was found between ACR TI-RADS and CNB results (ρ = 0.451; *p* < 0.001), indicating that higher ACR TI-RADS categories are associated with higher CNB scores, as shown in Figure 7.

A statistically significant positive correlation was observed between FNAB and CNB findings (ρ = 0.333; *p* = 0.002), indicating that higher FNAB scores are associated with higher CNB scores. When nodules with non-conclusive FNAB results (Bethesda I, III, and IV) were analyzed separately, no statistically significant association was found between FNAB and CNB results (ρ = 0.123; *p* = 0.354).

In contrast, for nodules with conclusive FNAB results (Bethesda II, V, and VI), a strong positive and statistically significant correlation was observed between FNAB and CNB scores (ρ = 0.814; *p* < 0.001).

### 3.7. FNAB–CNB Paired Analysis

For further analysis, a total of 82 nodules with paired FNAB and CNB results—i.e., both methods performed on the same nodule—were included (McNemar test; *p* < 0.001) (Table 3). Among the paired outcomes, both biopsies were non-conclusive in 15 patients (18.3%), while both FNAB and CNB were conclusive in 18 patients (22.0%). The highest number of discordant results was observed in patients with non-conclusive FNAB and conclusive CNB, accounting for 44 cases (53.7%). Conversely, conclusive FNAB with non-conclusive CNB was recorded in 5 patients (6.1%), as shown in Table 4.

### 3.8. Comparison with Definitive Findings

A total of 46 nodules were classified as malignant based on definitive findings. For FNAB, non-conclusive results were approximately equally distributed between patients with benign (70.4%) and malignant findings (67.4%), as confirmed by repeat FNAB, CNB, or post-surgical histology. In contrast, for CNB, conclusive results were significantly more frequent in patients with malignant findings (92.0%) compared to those with benign findings (63.6%). Conversely, non-conclusive CNB results were significantly more common in benign nodules (36.4%) compared to malignant nodules (8.0%), as confirmed by repeat CNB or post-surgical histology.

FNAB diagnostic accuracy (Bethesda II as benign; Bethesda V–VI as malignant) was evaluated in relation to definitive findings. Sensitivity was 71.4% (95% CI: 41.9–91.6%), specificity 100% (95% CI: 79.4–100%), positive predictive value 100% (95% CI: 69.2–100%), and negative predictive value 80% (95% CI: 56.3–94.3%). FNAB correctly identified 16/16 benign and 10/14 malignant cases, while 4 malignant cases were misclassified as benign.

**Figure 8:**
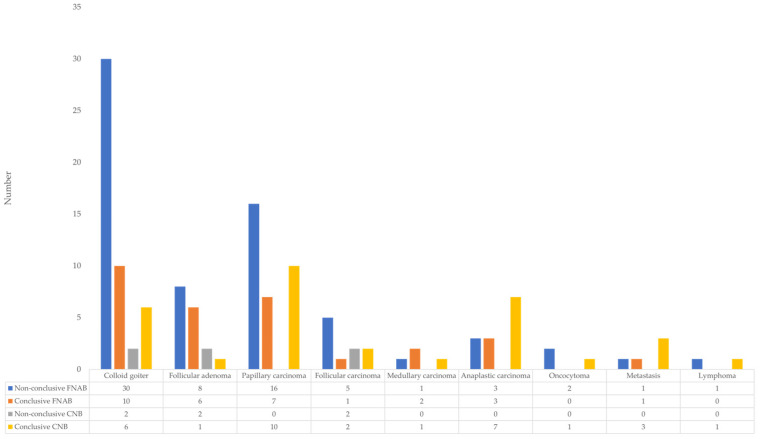
Distribution of patients with conclusive and non-conclusive FNAB and CNB results in relation to the type of benign and malignant findings, confirmed by definitive histopathological examination.

Figure 8 shows the distribution of various pathological findings in patients with thyroid nodules according to the type of biopsy and the conclusiveness of the result. The x-axis represents different diagnoses: colloid goiter, follicular adenoma, papillary carcinoma, follicular carcinoma, medullary carcinoma, anaplastic carcinoma, oncocytoma, metastases, and lymphoma. The y-axis shows the number of samples in each category. The most common non-conclusive findings with FNAB were in colloid goiter (30 cases) and papillary carcinoma (16 cases), whereas CNB showed a significantly lower number of non-conclusive results (at most 2 cases for colloid goiter, follicular adenoma, and follicular carcinoma). Similarly, CNB had a higher number of conclusive findings for papillary carcinoma (10 cases) and anaplastic carcinoma (7 cases) compared to FNAB. These data indicate a 100% performance of CNB for papillary, medullary, and anaplastic thyroid carcinomas, as well as for oncocytomas, metastatic changes in the thyroid, and lymphoproliferative disease, while a 50% performance was observed for follicular carcinoma. However, compared to FNAB, CNB achieved a higher rate of successful detection of malignant changes.

### 3.9. Complications

The frequency of minor complications differed significantly between FNAB and CNB procedures (*p* = 0.001). Minor complications were observed in 19 of 430 FNAB procedures (4.4%) and in 14 of 85 CNB procedures (16.5%). The frequency of major complications did not differ significantly between FNAB and CNB procedures (*p* = 1.000). Major complications occurred in 1 of 430 FNAB procedures (0.25%), whereas no major complications were recorded among the 85 CNB procedures (Table 5).

## 4. Discussion

In this study, we analyzed the diagnostic performance of FNAB and CNB in thyroid nodules, with particular emphasis on the frequency of non-conclusive findings and the role of CNB as a second-line approach. The results demonstrate a substantially lower proportion of indeterminate findings with CNB compared to FNAB, particularly in nodules that remained non-conclusive after initial cytology.

More than half of FNAB results in our cohort were classified as Bethesda I, III, or IV. This observation is consistent with the known limitations of cytological evaluation, especially in nodules with a follicular growth pattern, where architectural assessment is required to distinguish benign from malignant lesions [18,19]. In such scenarios, repeat FNAB is often performed; however, repeated cytology may still yield indeterminate results, prolonging the diagnostic process and occasionally leading to diagnostic surgery without definitive preoperative confirmation [20]. In contrast, CNB demonstrated a lower rate of non-conclusive findings and a significant association with definitive histopathological outcomes. The preservation of tissue architecture likely contributes to improved lesion characterization, particularly in cases of follicular-patterned nodules [21]. Similar findings have been reported in comparative and meta-analytic studies, where CNB showed higher diagnostic adequacy and reduced indeterminate rates when used after non-diagnostic FNAB [17,20]. Importantly, in our paired analysis, more than half of nodules with initially non-conclusive FNAB results were reclassified as conclusive following CNB, whereas the reverse situation was uncommon. This supports the selective use of CNB in cases where cytology does not provide sufficient diagnostic clarity.

Ultrasound risk stratification played an important complementary role. Hypoechogenicity, irregular margins, and punctate echogenic foci emerged as independent predictors of malignancy in multivariate analysis. The ultrasound features identified in our study—hypoechogenicity, irregular margins, and punctate echogenic foci—are well recognized in the literature as strong indicators of malignancy risk. In particular, these features form the cornerstone of structured risk stratification systems such as ACR TI-RADS, where they are consistently associated with higher categories and an increased likelihood of thyroid cancer. Notably, large multicenter analyses have demonstrated that punctate echogenic foci, corresponding to microcalcifications, are among the most specific predictors, while hypoechogenicity reflects underlying cellular density and reduced colloid content. Our findings reinforce the clinical relevance of these features in routine ultrasound practice and support their continued use in guiding biopsy decisions [22]. Although hypoechogenicity, irregular margins, and punctate echogenic foci were identified as independent predictors of malignancy in the multivariate analysis, the precision of these estimates is limited due to the relatively small number of outcome events and sample size, as reflected by the wide confidence intervals. Therefore, these findings should be interpreted with caution, and larger studies are needed to confirm the magnitude of these associations. These findings align with established ultrasound risk models and reinforce the importance of integrating imaging features with biopsy results [4,7]. In nodules with higher TI-RADS categories and persistent indeterminate cytology, CNB may represent a rational second-line diagnostic option.

The comparison of biopsy results with definitive outcomes further highlighted the difference between methods. No cases of follicular tumor of uncertain malignant potential (FT-UMP), noninvasive follicular thyroid neoplasm with papillary-like nuclear features NIFTP, or well-differentiated tumor of uncertain malignant potential (WDT-UMP) were identified among the biopsied nodules included in this study, which may reflect the relatively low prevalence of these borderline entities in routine clinical practice. While FNAB showed limited discriminatory capacity between benign and malignant lesions in non-conclusive categories, CNB demonstrated a statistically significant distinction [23,24]. These findings are in line with previous reports indicating that CNB may provide improved diagnostic precision in selected clinical contexts [21,23]. However, FNAB retains a well-established role in clearly benign (Bethesda II) and clearly malignant (Bethesda V–VI) categories, where cytomorphological assessment is generally sufficient for clinical decision-making [9,23]. The reported risk of malignancy (ROM) for Bethesda III (AUS) is approximately 22% (range 13–30%), reflecting the heterogeneity of this category and the associated challenges in clinical decision-making [16]. Regarding tissue sampling, our results align with previously reported data emphasizing the complementary roles of FNAB and CNB in thyroid nodule evaluation. In accordance with current guidelines, fine-needle aspiration biopsy (FNAB) remains the first-line diagnostic method for the evaluation of thyroid nodules, while core needle biopsy (CNB) is considered a complementary technique rather than a replacement [25]. CNB is particularly recommended in cases of non-diagnostic or indeterminate FNAB results, as well as in nodules with specific clinical or imaging suspicion, such as lymphoma, anaplastic carcinoma, metastatic lesions, or nodules with macrocalcifications. The utilization of CNB varies across different healthcare systems, with more frequent use reported in certain regions, particularly in East Asia, reflecting differences in clinical practice patterns and resource availability [26].

In our setting, the use of CNB was selective and guided by clinical and ultrasound findings, which is consistent with previously published recommendations [27]. Additionally, the lack of routine molecular testing in our healthcare system may further influence the diagnostic pathway and contribute to a greater reliance on tissue-based diagnostic methods in selected cases. Therefore, CNB should be interpreted as a complementary tool within a structured, individualized diagnostic approach, rather than as a substitute for FNAB.

While FNAB remains the first-line diagnostic tool due to its simplicity and safety profile, its limitations in indeterminate or non-diagnostic cases are well documented. In contrast, CNB has been shown to provide improved architectural detail and higher diagnostic yield in selected scenarios, particularly in lesions suspected of lymphoma, anaplastic carcinoma, or metastases. Our findings support a tailored approach, in which CNB is not used routinely but rather strategically, based on clinical and imaging suspicion [28,29,30,31].

With respect to safety, both procedures demonstrated acceptable profiles. Although minor complications were more frequent following CNB, all were self-limited and did not require additional intervention. No increase in major complications was observed, consistent with previously published data [21]. These findings suggest that, when performed under ultrasound guidance and with appropriate technique, CNB can be safely incorporated into the diagnostic workflow.

An advanced literature search using the terms “FNAB,” “CNB,” “thyroid nodules,” and “nonconclusive cytology” (2016–2026) identified a limited number of studies directly addressing this specific clinical scenario. This underscores the ongoing need for structured comparative analyses evaluating the role of CNB after indeterminate cytology.

This study has several limitations. It was conducted at a single center, and all procedures were performed by a single experienced radiologist, with pathological evaluation by a single pathologist. The application of the Bethesda classification system to CNB specimens, although previously described in the literature [16,18], represents a methodological adaptation, as the system was originally developed for cytological assessment. Additionally, the sample size of the CNB subgroup was smaller than that of FNAB, and cost-effectiveness, procedure duration, and patient-reported outcomes were not evaluated. The selection of patients for CNB was not randomized, and the decision to perform CNB as a first- or second-line procedure was based on clinical and ultrasound assessment, which may have introduced selection bias. Additionally, the relatively small number of first-line CNB procedures reflects the initial implementation of this technique in our center. The relatively small number of malignant cases (*n* = 46) in relation to the number of predictors included in the multivariable model may have resulted in limited model stability, as reflected by the wide confidence intervals. Therefore, these findings should be interpreted with caution. Future studies with larger sample sizes or the use of more parsimonious models or penalized regression methods may provide more stable estimates. Therefore, these results should be interpreted with caution.

Future research should include prospective, multicenter studies with standardized diagnostic algorithms to further clarify the optimal positioning of CNB within thyroid nodule management. Comparative cost analyses, assessment of time to definitive diagnosis, and integration with emerging molecular and imaging-based tools may provide additional insight into individualized diagnostic strategies.

## 5. Conclusions

In this cohort, core needle biopsy was associated with a significantly lower rate of indeterminate findings compared to fine-needle aspiration biopsy and showed closer concordance with definitive histopathological outcomes, particularly in nodules with initially non-conclusive cytology. Paired analysis confirmed that CNB frequently provides diagnostic clarification in cases where FNAB results remain indeterminate. Although FNAB remains an appropriate first-line diagnostic tool due to its simplicity and safety profile, selective implementation of CNB as a second-line procedure, especially in nodules with higher-risk ultrasound characteristics, may enhance diagnostic yield and reduce the need for repeat interventions. These findings support a structured, algorithm-based integration of CNB into the diagnostic pathway of thyroid nodules rather than its routine use as a primary diagnostic modality.

## Figures and Tables

**Table 1:** Ultrasound characteristics of nodules.

Ultrasound Characteristics	*N* (%)
**Nodule Composition**	
**Cystic or almost completely cystic**	9 (2.1)
**Spongiform**	3 (0.7)
**Solid-cystic**	69 (15.9)
**Solid**	352 (81.1)
**Echogenicity**	
**Anechoic**	9 (2.1)
**Hyperechoic**	81 (18.7)
**Isoechoic**	157 (36.2)
**Hypoechoic**	171 (39.5)
**Markedly hypoechoic**	15 (3.5)
**Margins**	
**Well-defined, smooth**	351 (81.3)
**Poorly defined**	3 (0.7)
**Lobulated or irregular**	69 (15.9)
**Extrathyroidal extension**	9 (2.1)
**Orientation**	
**Wider-than-tall (horizontal)**	297 (68.4)
**Taller-than-wide (vertical)**	135 (31.1)
**Echogenic Foci**	
**None**	223 (51.4)
**Large comet-tail artifacts**	18 (4.1)
**Macrocalcifications**	73 (16.8)
**Peripheral calcifications**	5 (1.2)
**Annular (rim) calcifications**	9 (2.1)
**Punctate echogenic foci**	103 (23.7)

**Table 2:** Univariate and multivariate logistic regression analyses of sonographic predictors of thyroid malignancy.

Parameters	Univariate Analysis			Multivariate Analysis		
	OR	95% CI	*p*	OR	95% CI	*p*
Nodule size	1.023	0.997–1.049	0.084	0.994	0.921–1.072	0.869
Echogenicity						
Hyper-/Isoechoic	ref.	ref.		ref.	ref.	
Hypoechoic/Markedly hypoechoic	6.632	2.707–16.246	0.000	22.321	1.304–382.101	0.032
Margins						
Well-defined, smooth						
Lobulated/Irregular/Extrathyroidal extension	4.033	1.643–9.899	0.002	14.606	1.009–211.422	0.049
Echogenic foci						
None	ref.	ref.		ref.	ref.	
Punctate echogenic foci	3.321	1.317–8.376	0.011	18.562	1.722–200.033	0.016
Other foci	2.460	0.819–7.389	0.109	8.601	0.283–261.585	0.217

**Table 3:** Comparison of FNAB and core biopsy according to definitive findings.

Characteristics		Definitive Finding—Benign *N* (%)	Definitive Finding—Malignant *N* (%)	*p*
**FNAB**	Non-conclusive	38 (70.4)	29 (67.4)	0.827
	Conclusive	16 (29.6)	14 (32.6)	
**CNB**	Non-conclusive	4 (36.4)	2 (8.0)	0.035
	Conclusive	7 (63.6)	23 (92.0)	

Definitive findings were established based on histopathological examination (when available), CNB results, and/or clinical follow-up.

**Table 4:** Analysis of FNAB and CNB outcomes in paired samples.

Characteristics		CNB	
		Non-Conclusive *N* (%)	Conclusive *N* (%)
**FNAB**	Non-conclusive	15 (75.0)	44 (71.0)
	Conclusive	5 (25.0)	18 (29.0)

McNemar test, *p* < 0.001.

**Table 5:** Frequency of minor and major complications after FNAB and CNB procedures.

	FNAB *N* (%)	CNB *N* (%)
Minor complication		
YES	19 (4.4)	14 (16.5)
NO	411 (95.6)	71 (83.5)
Major complication		
YES	1 (0.25)	0 (0.0)
NO	429 (99.75)	85 (100.00)

## Data Availability

The data presented in this study are available on request from the corresponding author due to privacy.

## Abbreviations

The following abbreviations are used in this manuscript:

TN	thyroid nodule
US	ultrasound
ACR TI-RADS	The American College of Radiology Thyroid Imaging Reporting and Data System
FNAB	fine-needle aspiration biopsy
CNB	core needle biopsy
FT-UMP	follicular tumor of uncertain malignant potential
NIFTP	noninvasive follicular thyroid neoplasm with papillary-like nuclear features
WDT-UMP	well-differentiated tumor of uncertain malignant potential
